# Repeated administration of imipramine modifies GABAergic transmission in rat frontal cortex

**DOI:** 10.1007/s00702-012-0919-3

**Published:** 2012-11-21

**Authors:** Joanna Wabno, Grzegorz Hess

**Affiliations:** 1 Department of Physiology, Institute of Pharmacology, Polish Academy of Sciences, Smetna 12, 31-343 Kraków, Poland; 2 Institute of Zoology, Jagiellonian University, Gronostajowa 9, 30-387 Kraków, Poland

**Keywords:** Tricyclic antidepressant, GABA_A_ receptor, Pyramidal neuron, Spontaneous IPSCs

## Abstract

Alterations in the functions of brain gamma-aminobutyric acid (GABA) inhibitory system and a distortion in the balance between excitatory and inhibitory synaptic transmission have been hypothesized to be possible causes of mood disorders. Experimental evidence points to modifications of GABAergic transmission as a result of prolonged treatment with antidepressant drugs, however, the influence of the tricyclic antidepressant imipramine on inhibitory synaptic transmission in the rat cerebral cortex has not yet been investigated. Therefore, in the present study the effects of single and repeated administration of imipramine were evaluated ex vivo in slices of the rat frontal cortex using electrophysiological approach. In slices prepared 2 days after the last drug administration from animals receiving imipramine for 14 days (dose 10 mg/kg p.o., twice daily) the mean frequency of spontaneous inhibitory postsynaptic currents (sIPSCs) recorded from layer II/III pyramidal neurons was decreased, while the mean amplitude of sIPSCs was increased. These effects were absent in slices obtained from rats which received imipramine once. Application of *N*,N′-dibenzhydrylethane-1,2-diamine dihydrochloride (AMN 082), a selective mGluR7 allosteric agonist, to the slice incubation medium resulted in a decrease in the mean frequency of sIPSCs in preparations obtained from repeated imipramine-treated animals, in contrast to slices originating from control rats where no AMN 082-induced effects were observed. Repeated imipramine treatment reduced protein density levels of the three tested GABA_A_ receptor subunits: *α*_1_, *β*_2_ and *γ*_2_. These data indicate that repeated treatment of normal rats with imipramine results in a modification of the release mechanism of GABA from presynaptic terminals and its modulation by mGluR7 receptors as well as in an alteration in GABA_A_ receptor subunit protein levels in the rat cerebral cortex.

## Introduction

A large proportion of the population affected by depression is not responding adequately to current treatments. It has been estimated that only one-third of patients respond to the first medication prescribed and that treatment-resistant depression may represent up to 50–70 % of cases (reviewed in: Duric and Duman 2012; Jenkins and Goldner 2012). Despite many years of research focusing on the efficacy of antidepressant drugs, the underlying mechanisms remain incompletely understood. At the cellular level, the primary effects of administration of a majority of currently used antidepressants seem to involve modulation of monoamine neurotransmission (reviewed in: Slattery et al. 2004; López-Muñoz and Alamo 2009; Murrough and Charney 2012). However, growing experimental evidence points to other effects of prolonged treatment with antidepressants, in particular to modifications of excitatory and inhibitory transmission mediated by glutamate and gamma-aminobutyric acid (GABA), respectively. These views are consistent with the data documenting alterations in the two brain amino acid neurotransmitter systems and a distortion in the balance between them, as possible causes of mood disorders (Sanacora et al. 2004; reviewed in: Skolnick et al. 2009; Croarkin et al. 2011; Hashimoto 2011; Luscher et al. 2011). Regarding the glutamatergic system, it has been shown that depressive disorders are accompanied by alterations in ionotropic AMPA and NMDA receptor-mediated synaptic transmission (reviewed in: Bleakman et al. 2007; Pittenger et al. 2007; Sanacora et al. 2012) and by altered levels of metabotropic glutamate receptors (Feyissa et al. 2010). Preliminary data indicate that symptoms of depression may be alleviated by treatments with drugs inhibiting the release of glutamate, lamotrigine and riluzole, as well as with the NMDA receptor antagonist, ketamine (Berman et al. 2000; Carlson et al. 2006; Zarate et al. 2010; Duman and Voleti 2012; Mathew et al. 2012).

Less is known about postulated depression-related modifications of the GABAergic system. Several studies did not find difference in the level of GABA in the cerebrospinal fluid of depressed patients in comparison with normal subjects but some researchers reported a reduction of the level of GABA in patients suffering from depression (reviewed in: Tunnicliff and Malatynska 2003, Croarkin et al. 2011, Luscher et al. 2011). Proton magnetic resonance spectroscopy provided evidence for a reduced level of GABA in certain cerebral cortical areas of depressed patients (Sanacora et al. 1999; Price et al. 2009). Major depression has also been shown to be accompanied by upregulation of several GABA_A_ receptor subunits (Choudary et al. 2005). These data are consistent with results obtained from animal models of human depression. For example in the olfactory bulbectomized rats the density of frontal cortical GABA_A_ receptors is decreased and this effect is reversed by a chronic treatment with antidepressants, including desipramine, an active metabolite of imipramine (Dennis et al. 1994). A depressive-like behavior has been found to characterize GABA_A_ receptor *γ*_2_ subunit-deficient mice (Shen et al. 2010). A decrease in the level of GABA, among other changes, has been found to occur in the prefrontal cortex of rats subjected to the chronic mild stress and chronic social defeat stress (Venzala et al. 2012). Chronic mild stress-related dysfunction of the GABAergic transmission was also revealed in the rat dentate gyrus (Holm et al. 2011).

Imipramine, a prototypic tricyclic antidepressant, is regarded one of the most effective drugs and is still used to treat severe cases of depression although it causes unwanted side effects (reviewed in: López-Muñoz and Alamo 2009). Imipramine acts as an inhibitor of serotonin and norepinephrine reuptake (reviewed in: Schloss and Williams 1998; Slattery et al. 2004). After the administration imipramine is rapidly metabolized with the formation of desipramine and other active hydroxy metabolites (Daniel et al. 1981; Potter et al. 1982; Gram 1988). A complex picture of changes in the expression pattern of numerous genes has been found to result from the treatment of rats with imipramine (Palotás et al. 2004) but one important outcome of repeated administration of imipramine involves a reduction in radioligand binding to *N*-methyl-d-aspartate (NMDA) receptors in the cerebral cortex suggestive of a suppression of glutamatergic transmission (Nowak et al. 1993). We have previously shown using electrophysiological recording techniques that repeated imipramine treatment of rats results in an attenuation of the release of glutamate from presynaptic terminals and an alteration in the reactivity of postsynaptic ionotropic glutamate receptors in the frontal cortex (Bobula et al. 2003; Bobula and Hess 2008; Tokarski et al. 2008), However, the influence of imipramine on inhibitory synaptic transmission in the rat cortex has not yet been investigated. Therefore, in the present study we aimed at finding the effects of repeated imipramine administration on spontaneous inhibitory postsynaptic currents (sIPSCs) recorded from frontal cortical pyramidal cells and on the protein amount of selected subunits of the GABA_A_ receptor. We chose *α*_1_, *β*_2_ and *γ*_2_ subunits since GABA_A_ receptors containing these subunits predominate in the cortex (Johnstone 2005; Luscher and Keller 2004; Skilbeck et al. 2010).

It has been reported that the major depressive disorder and anxiety disorders might be accompanied by alterations in the gene coding the metabotropic glutamate receptor 7 (mGluR7; reviewed by: Hamilton 2011), which is widely distributed in the human brain, including the frontal cortex (Makoff et al. 1996). This receptor appears to be located predominantly on presynaptic nerve terminals where it modulates the release of GABA and glutamate (Summa et al. 2012, reviewed in: O’Connor et al. 2010). Its selective agonist, *N*,*N*′-dibenzhydrylethane-1,2-diamine dihydrochloride (AMN 082), has been shown to induce anxiolytic-like and antidepressant-like effects in animal models (reviewed in: O’Connor et al. 2010; Sukoff Rizzo et al. 2011). In order to get insight into the influence of repeated imipramine treatment on mGluR7 receptor-dependent modulation of GABA release we tested the effects of the application of AMN 082 on the frequency of sIPSCs.

## Materials and methods

### Animals

Experimental procedures were approved by the Animal Care and Use Committee at the Institute of Pharmacology, Polish Academy of Sciences, and were carried out in accordance with the European Community guidelines for the use of experimental animals and national law. Male Wistar rats, weighing approx. 120 g at the beginning of the experiment, were obtained from Charles River, Germany. Animals were housed in groups and maintained on a 12 h light/dark schedule with standard food and tap water available ad libitum.

### Imipramine treatment and slice preparation

Two experimental and two control groups, each consisting of 8 rats, were investigated. Imipramine (Sigma-Aldrich), dissolved in water, was administered per os by using the gavage technique (dose 10 mg/kg, volume 2 ml/kg) either once (termed: single administration) or twice daily for 14 days (termed: repeated administration; Maj et al. 1982). The rats of the two control groups received water either once or twice daily for 14 days but otherwise they were handled identically and were investigated concurrently with imipramine-treated animals. It has earlier been shown that this procedure of drug administration per se does not influence the outcome of experiments (Bobula et al. 2003; Zahorodna et al. 2006).

Two days after the last imipramine administration rats were decapitated, their brains were quickly removed and placed in an ice-cold artificial cerebrospinal fluid (aCSF) containing (in mM): 130 NaCl, 5 KCl, 2.5 CaCl_2_, 1.3 MgSO_4_, 1.25 KH_2_PO_4_, 26 NaHCO_3_, 10 d-glucose and bubbled with the mixture of 95 % O_2_–5 % CO_2_. Frontal cortex slices (420 μm thick) from one hemisphere were cut in the coronal plane using a vibrating microtome (Leica VT1000). Slices were stored submerged in aCSF at 32 ± 0.5 °C and then used for electrophysiological experiments. The other brain hemisphere was placed on the dry ice and stored at −80 °C for later biochemical analysis.

### Whole-cell recording and analysis of sIPSCs

A slice was placed in the recording chamber and superfused at 3 ml/min with warm (32 ± 0.5 °C), modified aCSF of the following composition (in mM): 132 NaCl, 2 KCl, 1.25 KH_2_PO_4_, 26 NaHCO_3_, 1.3 MgSO_4_, 2.5 CaCl_2_, and 10 d-glucose 10, bubbled with 95 %O_2_–5 %CO_2_. Neurons were visualized using Zeiss Axioskop upright microscope using Nomarski optics, a 40× water immersion lens and an infrared camera (Tokarski et al. 2008). The neurons were sampled from sites located approx. 2 mm lateral to the midline and approx. 0.3 mm below the pial surface (layer II/III). Patch pipettes were pulled from borosilicate glass capillaries (Clark Electromedical Instruments) using Sutter Instrument P97 puller. The pipette solution contained (in mM): 130 K-gluconate, 5 NaCl, 0.3 CaCl_2_, 2 MgCl_2_, 10 HEPES, 5 Na_2_-ATP, 0.4 Na-GTP, and 1 EGTA. Osmolarity and pH were adjusted to 290 mOsm and 7.2, respectively. Pipettes had open tip resistance of approx. 6 MΩ. Signals were recorded using the SEC 05LX amplifier (NPI), filtered at 2 kHz and digitized at 20 kHz using Digidata 1322A interface and Clampex 9.2 software (Molecular Devices). Pyramidal cells were identified by the shape of the soma and the presence of a prominent apical dendrite as well as the regular spiking pattern showing adaptation in response to a depolarizing current pulse in the current clamp mode.

The neurons were voltage-clamped at 0 mV and sIPSCs were recorded for 8 min (Tokarski et al. 2007). Spontaneous IPSCs were detected off-line and analyzed using Mini Analysis software (Synaptosoft). Data were accepted for analysis when the access resistance ranged between 15 and 18 MΩ and it was stable (<25 % change) during recording. In part of experiments, after recording of sIPSCs for 4 min slices were perfused with the modified aCSF containing 1 μM AMN 082 (Sigma-Aldrich), a selective mGluR7 allosteric agonist. 10 min after the introduction of AMN 082 sIPSCs were again recorded for 4 min. In a subset of slices, 0.5 μM tetrodotoxin (TTX, Sigma-Aldrich) and bicuculline methiodide (10 μM, Tocris Bioscience) were added to the modified aCSF.

### Western blotting

Tissue samples were homogenized in ice-cold RIPA buffer (Sigma-Aldrich) containing phenylmethane-sulfonyl fluoride, sodium orthovanadate, protease inhibitor cocktail, phosphatase inhibitor cocktail 1, phosphatase inhibitor cocktail 2 and 5 % glycerol. The homogenate was centrifuged at 14,000 rpm for 20 min at 4 °C to obtain the crude membrane fraction. Protein concentration was determined using the BCA protein assay kit (Pierce Biotechnology, Inc.). A standard curve was generated and then absorbance of samples was measured with the Multiscan Spectrum Microplat Spectrophotometer (Thermo Labsystems). Supernatant form all samples was diluted with RIPA buffer to obtain the same protein concentration in 150 μl of the solution. Samples were boiled for 7 min at 99 °C in Laemmli (Bio-Rad) sample buffer with 2-mercaptoethanol (Sigma-Aldrich). To separate proteins, sodium dodecyl sulfate-polyacrylamide gel electrophoresis (SDS-PAGE) was performed first on 4 % upper gels (90 V, 30 min) to condense samples and then on 7.5 % lower gels (145 V, 60 min) with the Mini Protean Tetra Cell apparatus (Bio-Rad). Next, proteins were transferred to nitrocellulose membranes (Bio-Rad) by electrophoresis in transfer buffer containing 20 % methanol, at 90 V, during 1 h (Mini Protean Tetra Cell; Bio-Rad). Following transfer, the membranes were washed in Tris-buffered saline (TBS; 2×, 10 min) and immersed in blocking solution consisting of 3 % albumin from bovine serum (Sigma-Aldrich) and TBST, for 60 min at room temperature. Afterwards, the membranes were incubated with an affinity-purified polyclonal antibodies against the following GABA_A_ receptor subunits: *α*_1_ (1:200, from goat; Santa Cruz Biotechnology, Inc.), *β*_2_ and *γ*_2_ (1:1,000, from rabbit; Millipore) in 1 % albumin—TBST at 4 °C overnight. On the next day the membranes were incubated for 60 min at room temperatures, then washed two times for 10 min in TBST and two times for 10 min in 1 % albumin solution. The membranes were incubated with horseradish peroxidase-conjugated secondary antibody (1:2,000, Vector Laboratories) in 1 % albumin—TBST, for 60 min at room temperature. The secondary antibody used for the *α*_1_ subunit was an anti-goat antibody, whereas the secondary antibody used for the *β*_2_ and *γ*_2_ subunits was an anti-rabbit antibody. The membranes were washed four times for 15 min in TBST.

Proteins were detected using chemiluminescent detection method (Immun-Star HRP). The membranes were exposed to luminol for 4 min. Luminol was activated with an oxidant (horseradish peroxidase) and irradiation and pictures were made with FujiLas 4000. The signal of the specific band was expressed as the optical density level. Results were standardized and presented as percentage change from the control value. Signals obtained in the same membrane but area different than bands were subtracted for background correction (Kharlamov et al. 2008; Leung et al. 2002).

### Statistics

All results are expressed as mean ± SEM. Statistical analysis involved pairs of comparisons between experimental versus control groups and it was carried out using Student’s *t* test for sIPSCs and Western blotting data, and paired *t* test—in experiments where AMN 082 was applied.

## Results

**Fig. 1:**
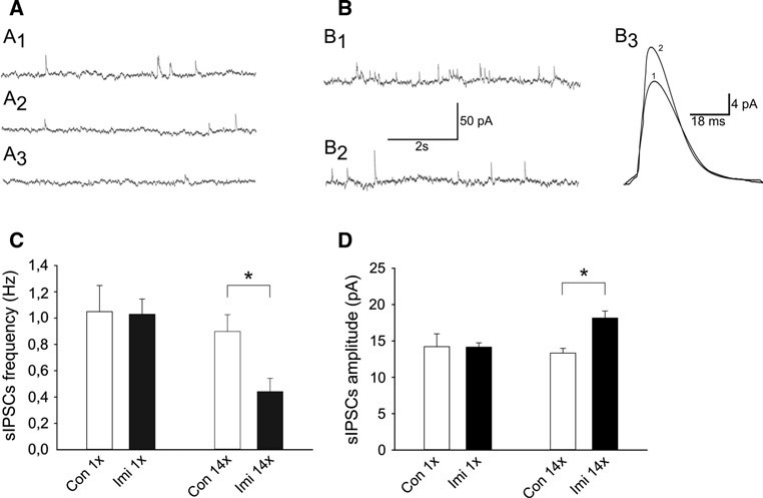
Repeated imipramine-induced changes in the mean frequency and the mean amplitude of spontaneous IPSCs in layer II/III pyramidal cells. An example of recording from an individual neuron in a slice prepared from control, untreated animal in the aCSF (**a**_1_) and after addition of TTX (**a**_2_) as well as bicuculline methiodide (**a**_3_). Upward deflections represent individual sIPSCs. **b**_1_ An example of recording from another control cell and **b**_2_ from the cell originating from repeated imipramine-treated rat. **b**_3_ Superposition of averaged sIPSCs from the raw recordings whose parts are shown in **b**_1_ and **b**_2_ (*1* control; *2* repeated imipramine-treated). **c** Mean (±SEM) frequency of sIPSCs in control neurons (Con) and neurons from single (Imi 1×) and repeated imipramine (Imi 14×)-treated animals. **d** Mean (±SEM) amplitude of sIPSCs. Abbreviations as in **c**. *p < 0.05, Student’s *t* test

**Fig. 2:**
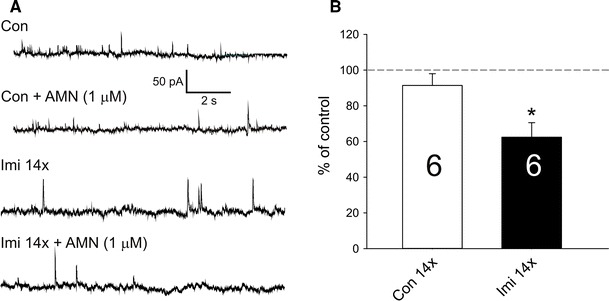
Effect of 1 μM AMN 082 on the frequency of sIPSCs. **a** Examples of sIPSCs (upward deflections) recording from slices obtained from control (Con) and repeated imipramine-treated (Imi 14×) rats and the effects of AMN 082 (AMN) addition to aCSF. **b** Mean (±SEM) frequency of sIPSCs after AMN 082 application in the control (Con) and repeated imipramine-treated (Imi 14×) group. *Number on bars* represent numbers of cells in each group; *p < 0.05, paired *t* test

The cells included in the analysis exhibited a regular spiking firing pattern in response to a depolarizing current pulse and no spontaneous spiking activity (not shown). At the holding potential of 0 mV sIPSCs were recorded as inward currents (Figs. 1a, b; 2a). To test what fraction of spontaneous IPSCs represented spiking activity-independent miniature IPSCs (mIPSCs) in a sample of five cells spontaneous events were recorded before and after addition of 0.5 μM TTX to block action potentials. In the presence of TTX the mean frequency of IPSCs decreased to 86.2 ± 9.8 % of baseline (Fig. 1a_1_, a_2_), but this change was not statistically significant (*p* > 0.05) which indicates that the majority of recorded spontaneous events corresponded to mIPSCs. After addition of 10 μM bicuculline methiodide spontaneous events disappeared (Fig. 1a_3_) confirming that they represented GABA_A_ receptor-dependent currents.

There was no significant difference between control neurons and cells in slices originating from imipramine-treated rats either in the resting membrane potential or in the input resistance (Table 1). There was also no difference in the mean frequency and the mean amplitude of sIPSCs in neurons originating from control animals and those which received a single dose of imipramine (Fig. 1c, d). In contrast, in slices prepared from repeated imipramine-treated rats the mean sIPSCs frequency was decreased and the mean sIPSCs amplitude—increased, in comparison to control preparations (Fig. 1c, d). There was no significant difference between control neurons and cells in slices originating from either single or repeated imipramine-treated rats in the parameters characterizing the kinetics of sIPSCs: the rise time and the decay time constant (Table 2).

**Table 1:** Basic parameters of recorded cells

Group	*V*_m_ (mV)	*R*_m_ (MΩ)	*n*
Con 1×	−70.40 ± 1.34	39.01 ± 3.25	9
Imi 1×	−69.15 ± 1.16	37.30 ± 5.53	9
Con 14×	−70.00 ± 1.18	40.15 ± 2.81	13
Imi 14×	−71.07 ± 1.09	37.93 ± 1.80	13

Data are presented as the mean ± SEM. Differences between values for neurons in experimental and control groups are not significant (*p* > 0.05)

*V*_*m*_ resting membrane potential, *R*_*m*_ input resistance, *Con* control neurons, *Imi* neurons originating from animals receiving imipramine once (1×) or for 14 days (14×)

**Table 2:** Rise time and decay time constant (*τ*) of averaged sIPSCs

Group	Rise time (ms)	*τ* (ms)	*n*
Con 1×	2.90 ± 0.14	12.33 ± 1.52	9
Imi 1×	2.79 ± 0.10	11.27 ± 0.79	9
Con 14×	3.05 ± 0.26	11.33 ± 0.81	13
Imi 14×	3.14 ± 0.11	12.46 ± 1.10	13

Data are presented as the mean ± SEM. Rise time was measured between 10 and 90 % of maximum amplitude. Decay time constant (*τ*) was determined as the time required for the current to decay to 36.8 % of its maximum value. Differences between values for neurons in experimental and control groups are not significant (*p* > 0.05)

*Con* control neurons, *Imi* neurons originating from animals receiving imipramine once (1×) or for 14 days (14×)

While in the control group the application of AMN 082 to the aCSF induced no significant change in the mean frequency of sIPSCs, in cells originating from repeated imipramine-treated animals AMN 082 application reduced the mean frequency of sIPSCs (Fig. 2a, b).

**Fig. 3:**
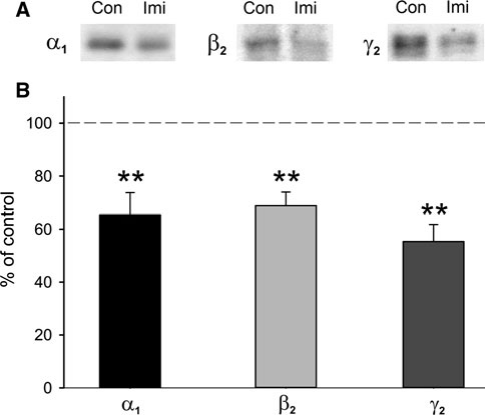
Western blot analysis of GABA_A_ receptor subunits proteins. **a** Computerized scans of representative Western immunoblots illustrating the ratio of *α*_1_ (51 kDa), *β*_2_ (55 kDa) and *γ*_2_ (46 kDa) subunit protein bands in repeated imipramine-treated (Imi) and control (Con) groups. **b** *Bars* representing summary results of densitometric analysis of *α*_1_, *β*_2_ and *γ*_2_ subunit proteins from the cortex of repeated imipramine-treated rats (mean ± SEM), relative to the tissue from control rats. **p < 0.01, Student’s *t* test. In each *bar* *n* = 6–8

Western blot analysis revealed that repeated administration of imipramine reduced protein density levels of all three tested GABA_A_ receptor subunits: *α*_1_, *β*_2_ and *γ*_2_ (Fig. 3b).

## Discussion

We have found that a single dose of imipramine (10 mg/kg) did not affect sIPSCs, whereas the 2 week-long treatment resulted in a decrease in the mean frequency and an increase in the mean amplitude of sIPSCs. We have not determined the plasma level of imipramine, however, the level of imipramine administered in a dose of 10 mg/kg either once or twice a day for 14 days has been measured in blood and brain tissue by Daniel et al. (1981). These investigators have found that within 1 h after a single intraperitoneal injection the concentration of imipramine in the plasma reaches about 0.5–0.6 μg/ml and then it decreases below the detection level within next 9 h. After chronic treatment the maximum level of imipramine after its last administration was higher (approx. 0.8 μg/ml) and the elimination of the drug was slowed down. The concentration of imipramine in the brain has been found to be about ten times higher than in blood and it has been concluded that during repeated administration imipramine, and its metabolite desipramine, are continuously present in the brain tissue at concentrations which inhibit noradrenaline and serotonin uptake. When imipramine is administered orally the desipramine/imipramine concentration ratio in the brain is higher due to the demethylation of imipramine in the liver (Maj et al. 1982).

While the amplitude of spontaneous postsynaptic currents is determined by a combination of pre- and postsynaptic factors, a decreased frequency of sIPSCs in principle reflects either reduced firing of presynaptic cells or decreased neurotransmitter release either due to a change in release probability and/or change in the number of release sites. Since only a non-significant decrease in sIPSCs frequency was observed after addition of TTX to aCSF in a subset of cells, demonstrating that most of the recorded spontaneous events corresponded to spike-independent mIPSCs, we consider the possibility that observed effects result from a change in spiking activity of presynaptic neurons unlikely. Therefore, a decrease in the frequency of sIPSCs most likely reflects repeated imipramine treatment-related modification of the mechanism of GABA release from presynaptic terminals. We have previously shown that the same treatment results in an approximately twofold decrease in the frequency of glutamatergic spontaneous excitatory postsynaptic currents (sEPSCs) in rat frontal cortical pyramidal cells (Tokarski et al. 2008). Thus, in the frontal cortex repeated imipramine treatment appears to exert a similar effect on the release from presynaptic terminals of both the excitatory and the inhibitory amino acid.

The available data on imipramine-induced modifications of neurotransmitter release mechanisms are scarce. Chronic imipramine induced a decrease of potassium-stimulated glutamate outflow in rat prefrontal cortex (Michael-Titus et al. 2000). In the hippocampus, treatment with antidepressant drugs, including desipramine, reduced the protein–protein interaction between syntaxin 1 and *α*-calcium/calmodulin-dependent protein kinase II, thought to promote glutamate release, and simultaneously increased the interaction between syntaxin and Munc-18, thought to reduce release (Bonanno et al. 2005). It is conceivable that similar mechanisms might be involved in GABAergic synapses in the frontal cortex but it should be noted that these authors found that while the tested drugs reduced the release of glutamate in the hippocampus, GABA release remained unchanged.

One possible mechanism of the modification of the release of GABA relates to alterations in modulatory effects imposed by presynaptic mGluR7 receptors. The present results demonstrate that while under control experimental conditions 1 μM AMN 082 did not affect the frequency of sIPSCs, in slices obtained from repeated imipramine-treated rats the mGluR7 receptor agonist decreased the mean sIPSCs frequency. Activation of mGluR7 receptors inhibits neurotransmitter release through a reduction in intracellular cAMP level and a decrease in presynaptic Ca^2+^ influx (Millan et al. 2002). The inhibitory effect of AMN 082 on the release of GABA from presynaptic terminals is dose-dependent (Summa et al. 2012). Since it has been demonstrated that imipramine, but not the selective serotonin reuptake inhibitor (SSRI), citalopram, administered repeatedly for 21 days produced no change in mGluR7 immunoreactivity in the rat cortex (Wieronska et al. 2007), we conclude that repeated imipramine appears to induce a functional sensitization of this system at the post-receptor level. It remains to be established whether other mechanisms contribute to the observed effect of repeated imipramine administration on presynaptic GABA release machinery.

Earlier research demonstrated that 21 days-long constant, subcutaneous infusion of imipramine did not produce changes in the level of mRNAs encoding GABA synthesizing enzymes, GAD65 and GAD67, the GABA membrane transporter GAT-1 (SLC6A1 according to the HUGO Gene Nomenclature Committee) as well as the catabolizing enzyme, GABA transaminase in the rat cortex (Lai et al. 1998). However, it cannot be excluded that the observed increase in the mean amplitude of sIPSCs is a result of an increased neurotransmitter content of synaptic vesicles. To our knowledge there is no available data for the level and the activity of the vesicular GABA transporter (VGAT/SLC32A1) after antidepressant treatments but, for example, the antipsychotic drug clozapine increases the amount of VGAT/SLC32A1 in rat frontal cortex (Bragina et al. 2007). On the other hand, rats with the genetic deletion of the serotonin transporter, expressing an anxious and depressive phenotype, demonstrated a.o. a reduced level of VGAT/SLC32A1 (Guidotti et al. 2012). In an animal model of stress and depression the expression level of vesicular glutamate transporter VGLUT1/SLC17A7 was reduced in the rat cortex (Zink et al. 2010). Moreover, decreased expression of VGLUT1/SLC17A7 results in an increased depressive-like behavior (Garcia-Garcia et al. 2009) and several antidepressant drugs, including desipramine, enhanced VGLUT1/SLC17A7 expression in the cortex (Moutsimilli et al. 2005). Thus, we hypothesize that in our experiments a similar effect on the vesicular transporter in GABAergic synapses might have been induced by imipramine.

We have found that repeated imipramine treatment resulted in a similar degree of a decrease in protein density levels of *α*_1_, *β*_2_ and *γ*_2_ subunits in rat frontal cortex. Since these subunits together build the most abundant GABA_A_ receptor subtype in the cortex (Johnstone 2005; Luscher and Keller 2004; Skilbeck et al. 2010), this result is consistent with the downregulation of this receptor subtype. The effects of imipramine on expression of GABA_A_ receptors in rat cortex have not been investigated before. In the brainstem, chronic imipramine induced an increase in the level of mRNAs of *α*_3_, *β*_1_ and *γ*_2_, but not other, subunits (Tanay et al. 2001). Differences in the experimental design and in the investigated brain area may account for this discrepancy. It is conceivable that the putative downregulation of GABA_A_ receptor subunits reflects a compensatory process related to a postulated increase in the neurotransmitter content of synaptic vesicles.

Summing up, these data show that imipramine, administered repeatedly, modifies the release of GABA from presynaptic terminals in a complex way, by reducing the mean frequency but increasing the mean amplitude of sIPSCs, and reduces protein amount of the tested GABA_A_ receptor subunits in the rat frontal cortex. These results do not easily fit with the current GABAergic hypothesis of depression which implicates the deficit in the function of the GABAergic system within the hippocampus and other brain regions in the pathophysiology of the illness and at least partial reversal of this deficit by antidepressants in human patients (reviewed in: Croarkin et al. 2011; Luscher et al. 2011). Also studies employing animal models of depression point to a reduction in the level of GABAergic markers and an enhancement of GABAergic transmission due to chronic treatment with antidepressants (Dennis et al. 1994; Shen et al. 2010; reviewed in: Luscher et al. 2011). However, it should be noted that the present work was conducted using normal, healthy animals. It has been shown that chronic treatment with the SSRI, fluoxetine, reduces GABAergic transmission in the visual cortex (Maya Vetencourt et al. 2008) and reduces evoked IPSCs in the prefrontal cortex of normal rats (Zhong and Yan 2004). Thus, it is conceivable that in rats representing one of the animal models of depression, for example subjected to a chronic stress, the effects of imipramine on GABAergic transmission in the frontal cortex might be different. This possibility remains to be investigated in future studies.
